# A reporter mouse for optical imaging of inflammation in *mdx* muscles

**DOI:** 10.1186/s13395-015-0042-x

**Published:** 2015-04-30

**Authors:** Leonel Martinez, Natalia V Ermolova, Tomo-O Ishikawa, David B Stout, Harvey R Herschman, Melissa J Spencer

**Affiliations:** Department of Neurology and Center for Duchenne Muscular Dystrophy, David Geffen School of Medicine, University of California Los Angeles, 635 Charles Young Dr. South, NRB Room 401, Los Angeles, CA 90095 USA; Department of Biological Chemistry, David Geffen School of Medicine, University of California Los Angeles, 341 Boyer Hall, 611 Charles E Young Dr. So, Los Angeles, CA 90095 USA; Department of Molecular and Medical Pharmacology, David Geffen School of Medicine, University of California Los Angeles, Los Angeles, CA 90095 USA; Center for Duchenne Muscular Dystrophy at UCLA, Los Angeles, CA 90095 USA; Molecular Biology Institute, UCLA, Los Angeles, CA 90095 USA; Present address: Genomics Business Department, Trans Genic Inc, Kumamoto, 862-0976 Japan

**Keywords:** *mdx*, Duchenne muscular dystrophy, Inflammation, Imaging, Reporter mouse, Immune cells, Cox2, Cyclooxygenase 2, Luciferase

## Abstract

**Background:**

Duchenne muscular dystrophy (DMD) is due to mutations in the gene coding for human *DMD*; DMD is characterized by progressive muscle degeneration, inflammation, fat accumulation, and fibrosis. The *mdx* mouse model of DMD lacks dystrophin protein and undergoes a predictable disease course. While this model has been a valuable resource for pre-clinical studies aiming to test therapeutic compounds, its utility is compromised by a lack of reliable biochemical tools to quantifiably assay muscle disease. Additionally, there are few non-invasive assays available to researchers for measuring early indicators of disease progression in *mdx* mice.

**Methods:**

*Mdx* mice were crossed to knock-in mice expressing luciferase from the Cox2 promoter. These reporter mice (*Cox2*^*FLuc/+*^*DMD*^*−/−*^) were created to serve as a tool for researchers to evaluate muscle inflammation. Luciferase expression was assayed by immunohistochemistry to insure that it correlated with muscle lesions. The luciferase signal was quantified by optical imaging and luciferase assays to verify that the signal correlated with muscle damage. As proof of principle, *Cox2*^*FLuc/+*^*DMD*^*−/−*^ mice were also treated with prednisolone to validate that a reduction in luciferase signal correlated with prednisone treatment.

**Results:**

In this investigation, a novel reporter mouse (*Cox2*^*FLuc/+*^*DMD*^*−/−*^ mice) was created and validated for non-invasive quantification of muscle inflammation *in vivo*. In this dystrophic mouse, luciferase is expressed from cyclooxygenase 2 (Cox2) expressing cells and bioluminescence is detected by optical imaging. Bioluminescence is significantly enhanced in damaged muscle of exercised *Cox2*^*FLuc/+*^*DMD*^*−/−*^ mice compared to non-exercised *Cox2*^*FLuc/+*^*DMD*^*+/+*^ mice. Moreover, the Cox2 bioluminescent signal is reduced in *Cox2*^*FLuc/+*^*DMD*^*−/−*^ mice in response to a course of steroid treatment. Reduction in bioluminescence is detectable prior to measurable therapy-elicited improvements in muscle strength, as assessed by traditional means. Biochemical assay of luciferase provides a second means to quantify muscle inflammation.

**Conclusions:**

The *Cox2*^*FLuc/+*^*DMD*^*−/−*^ mouse is a novel tool to evaluate the therapeutic benefits of drugs intended to target inflammatory aspects of dystrophic pathology. This mouse model will be a useful adjunct to traditional outcome measures in assessing potential therapeutic compounds.

## Background

Duchenne muscular dystrophy (DMD) is a disease characterized by muscle degeneration followed by inflammation, fatty infiltration, and fibrosis. In humans, the disease results from mutations in the *DMD* gene coding for dystrophin, a large cytoskeletal protein that links the actin cytoskeleton to the subsarcolemmal membrane via a complex called the dystrophin glycoprotein complex (DGC) [1-3]. In the absence of dystrophin, the entire DGC is lost from the membrane and recurrent cycles of muscle damage, inflammation, and repair ensue. While muscle regeneration partially compensates for necrosis, lost muscle is eventually replaced by fibrotic tissue.

The *mdx* mouse (C57BL/10ScSn-*Dmd*^*mdx*^/J), the genetic homologue for human DMD, is a commonly used animal model [4-8]. While *mdx* mice display a less severe phenotype compared to their human counterparts, pathology and muscle weaknesses are evident morphologically from 3 weeks of age. Subsequently, the muscles undergo a predictable course of disease. *Mdx* mice are weaker than age-matched wild-type mice when evaluated by a variety of muscle strength tests [9]. In addition, their serum creatine kinase remains elevated throughout their lives [10]. Thus, the *mdx* mouse has been a highly useful tool for pre-clinical studies to facilitate drug discovery for dystrophinopathies.

While the *mdx* mouse is useful for identification of pathogenic mechanisms, quantitative assessment of early disease features (for example, inflammation) is more difficult to evaluate in this model. This difficulty is due in part to the unpredictable location of muscle lesions, which can be asymmetric and focal [10]. Furthermore, since the sampling of a given muscle section is not always representative of the entire muscle, assessment of individual muscle sections may be inaccurate in evaluating the extent of muscle damage. Thus, models that enable high throughput, rapid, non-invasive, and longitudinal assessment of the efficacy of therapeutic compounds in mice would be a useful resource to muscular dystrophy researchers.

To address some of these concerns, we generated a knock-in mouse in which luciferase is expressed from the Cox2 gene (*PTGS2*) promoter (*Cox2*^*FLuc/+*^ mice) [11]. Cox2 is one of the enzymes participating in prostaglandin synthesis; it is induced in numerous cell types, particularly macrophages, which are the predominant immune cell type in dystrophic muscle lesions [12]. Furthermore, the appearance of macrophages in dystrophic muscle closely parallels active disease [13], suggesting that *Cox2*^*FLuc/*+^ mice may be a useful reporter to monitor disease progression in *mdx* mice (*DMD*^*−*^*/*^*−*^). After crossing the *Cox2*^*FLuc/+*^ mouse to the *DMD*^*−/−*^*mdx* background to create *Cox2*^*FLuc/+*^*DMD*^*−/−*^ mice, we show that dystrophic features of inflammation can be monitored non-invasively by bioluminescent optical imaging.

A major advantage to using non-invasive, whole-animal imaging systems for detection of muscle damage is the ability to obtain quantifiable assessments of the efficacy of therapeutic candidates. Currently, the primary method used by researchers to assess muscle damage in the dystrophies is histopathology. However, this approach is time consuming and is limited to analysis of only one or two sections of each muscle. Optical imaging systems can detect light from luciferase-expressing cells after injection of the substrate luciferin. In the presence of sufficient ATP, Mg, and luciferin, light is produced in amounts proportional to the amount of luciferase enzyme expressed. In appropriate circumstances, the light can be detected and quantified from living animals. The procedure is much less expensive than other methods of imaging, for example, magnetic resonance imaging or positron emission tomography. Furthermore, for luciferase-based reporters, the reporter gene signal is produced only at sites where the enzyme is expressed, unlike PET imaging where the unbound radioactive substrates/ligands must be cleared for the reporter gene-mediated signal-to-background contrast to be observed. Where applicable and appropriate, optical reporter gene imaging provides advantage of ease of use and low cost in carrying out longitudinal studies [14,15].

In this manuscript, we describe a novel reporter mouse, useful for assessing pharmacological treatments for DMD. The *Cox2*^*FLuc/+*^*DMD*^*−/−*^ mouse model shows sufficient sensitivity to both optically and biochemically quantify changes in damage mediated by steroid treatment. Thus, this mouse model is now an additional tool available to muscle researchers to evaluate potential new therapeutic compounds.

## Methods

### Animals

*Cox2*^*FLuc/+*^*DMD*^*−/−*^ mice were created by crossing the commonly used model of human DMD, the *mdx* mouse, (C57BL/10ScSn-*mdx*/J) with the cyclooxygenase2 (*Cox2*)-luciferase knock-in mouse (*Cox2*^*FLuc/+*^*BL/6)* created by our laboratory [11]. The *Cox2*-luciferase knock-in transgene was always maintained in the heterozygous state. The *Cox2* knock-in mouse was crossed to the C57BL/6 background five times and then crossed to *mdx* (C57BL/10ScSn-*Dmd*^*mdx*^/J, Jackson labs) to create *Cox2*^*FLuc/+*^*DMD*^*−/−*^ mice. Mice expressing the *Cox2*^*FLuc*^ allele on the wild-type *DMD*^*+/+*^ background (identified as *Cox2*^*FLuc/+*^*DMD*^*+/+*^) were used as controls to assess background signal. All animal work was conducted under protocols approved by the UCLA Animal Research Committee in the Office of Animal Research Oversight (protocol numbers ARC# 1998–078 and ARC#2009-029).

### Exercise

Mice used for imaging were given a running wheel at 3 weeks of age (InnoDome and InnoWheel, Bio Serv, Flemington, NJ). Wheels remained in the cages throughout the experiment. Running wheels were cleaned, autoclaved, and replaced weekly. In addition, 2 days prior to imaging, mice were exercised on an Eco 3/6 treadmill (Columbus Instruments, Columbus, OH) to exacerbate the dystrophy. *Cox2*^*FLuc/+*^*DMD*^*−/−*^ mice were subjected to 20 min of running at 19 m/min on a decline of 8°. *Cox2*^*FLuc/+*^*DMD*^*+/+*^ mice were not exercised so that basal levels of signal could be determined, independent of muscle damage.

### *In vivo* bioluminescent imaging

Bioluminescence was assessed using the Xenogen IVIS Imaging system (STTARR, Toronto, ON, Canada). One day prior to imaging, mouse legs were shaved. Mouse cages were warmed for 20 min on heating plates at 37°C, and temperature support was maintained during luciferin uptake and imaging. Mice were anesthetized and maintained on 1.5% to 2% isoflurane gas anesthesia in oxygen. After mice were weighed, they were injected intraperitoneally with D-luciferin (125 mg/kg) and immediately placed supine in a sealed imaging chamber. Images were acquired using medium binning and one-minute exposures at 15, 20, 25, and 30-minute time points after D-luciferin injection. Using the Living Image®4.0 software (Perkin Elmer, Waltham, MA), regions of interest were created around the hindlegs. Bioluminescence was quantified with Living Image software to determine max radiance (photons).

### Cardiotoxin-induced injury

To assess the ability of optical imaging to detect muscle damage, we induced acute muscle damage by cardiotoxin (CTX) injection (50 μl of 10 μM) to the gastrocnemius muscle of *Cox2*^*FLuc/+*^*DMD*^*+/+*^ mice [16]. The contralateral leg was not injected. Two days later, mice were assessed by optical imaging, as described above. At the completion of the imaging session, mice were given a second injection of luciferin and, after 10 min, the mice were sacrificed, the muscles were dissected, placed in a petri dish, and imaged *ex vivo*.

### Wire test

Mice were tested by wire test as previously described [12]. Briefly, mice were placed on a wire secured 2 ft above a safety net and allowed to use forelimbs and hindlimbs (but not their tail) to hang. Each mouse was subjected to five trials, with 1 minute of rest between trials. Hang time was recorded from the moment the experimenter placed the mouse onto the wire until the mouse fell onto the safety net. The five data points were averaged and normalized by body weight.

### Prednisolone steroid treatment

Female and male mice were treated with 0.75 mg/kg of prednisolone, 21-hemisuccinate sodium salt (Sigma, St Louis, MO) in sterile phosphate-buffered saline (PBS) [17]. Treated mice received intraperitoneal injections 5 days a week, beginning from 2 weeks of age until muscles were harvested. Prior to each injection, mice were weighed and the dosage was adjusted. Control mice received equal volumes of PBS via intraperitoneal injections.

### Immunohistochemistry

Immunohistochemistry was carried out on 10 μm frozen sections, as previously described [13]. Muscles to be assayed by immunohistochemistry were harvested and placed in OCT (Sakura Finetek, Torrance, CA, USA) then frozen in isopentane cooled by liquid nitrogen. Muscles were cross-sectioned (10 μm) and stained using the MOM kit (Vector Laboratories, Burlingame, CA, USA) and either the AEC Peroxidase Substrate Kit (Vector Laboratories) or using fluorophore-conjugated antibodies. Antibodies used were developmental myosin heavy chain (1:25, Leica Biosystems, Teban Gardens Crescent, Singapore), CD11b (1:50, BD PharMingen, San Diego, CA, USA), CD68 (1:50, BD PharMingen), and luciferase (1:25, Promega, Madison, WI, USA). Images were acquired using a compound microscope (Zeiss, Ontario, CA, USA), processed using AxioVision software (Zeiss), and displayed using Photoshop CS4 (Adobe, Mountain View, CA, USA).

### Luciferase assay

Luciferase assay of muscles *ex vivo* was performed using the Promega Luciferase Assay Kit (cat# E1500, Promega) according to the manufacturer’s recommendations with minor modification. In brief, 40 to 100 mg of frozen muscle was powdered with mortar and pestle in liquid nitrogen. Powder was transferred to 1.5 ml tubes, and 0.5 ml of Passive Lysis Buffer (PLB; Cat# E1941, Promega) was added to the tube. Extraction was performed for 1 h at 4°C with rotation followed by 3 cycles of freezing/thawing in liquid nitrogen or 30°C water bath. Samples were vortexed after each thawing cycle for 1 min at room temperature. Samples were then subjected to centrifugation at 10,000 g for 15 min at 4°C. Clarified supernatants were transferred to new tubes and stored at −80°C until used for luciferase assay and protein concentration measurements. For luciferase assay, 20 μl of sample were mixed with 100 μl of assay reagent and luciferase activity was recorded immediately using a Promega GloMax 2020 Luminometer. The amount of luciferase in the sample was calculated using the standard curve prepared with commercially available QuantiLum Recombinant Luciferase (cat# E1707, Promega). The amount of protein in the sample was measured using the 660 nm Protein assay kit (Pierce, cat#22660, Life Technologies, Grand Island, NY, USA) with bovine serum albumin (BSA) used as a standard. All measurements were performed in triplicate.

### Tissue extract preparation and western blot analysis

For western blot analysis, gastrocnemius muscles from *Cox2*^*FLuc/+*^ mice were homogenized in reducing sample buffer (80 mM Tris, pH 6.8, 0.1 M dithiothreitol, 2% SDS, and 10% glycerol with protease inhibitor cocktail (Sigma-Aldrich, St. Louis, MO, USA)) using a Dounce homogenizer. Protein extracts were boiled for 1 minute, placed on ice, and loaded to SDS PAGE (40 μg per well) followed by transfer to nitrocellulose membrane (100 V, 1 h, 4°C). As a positive control, 7.5 ng of QuantiLum recombinant luciferase (Promega, E1701) was used. Membranes were stained with Ponceau red and imaged and then blocked with 3% BSA and probed with anti-luciferase antibody (Promega, 1:1,000) followed by anti-rabbit IgG peroxidase conjugate (Sigma-Aldrich). Blots were developed using ChemiGlow West substrate (ProteinSimple, San Jose, CA, USA) and detected using an AlphaImager gel documentation system (formerly Alpha Innotech, now ProteinSimple). The actin band from the Ponceau stained membrane served as the loading control.

### Statistical analysis

For optical imaging, the four images that were acquired at 15, 20, 25, and 30 min were averaged. At each of these four time points, both legs of each animal were averaged and this number was used as the data point. Thus, each point for data analysis included both the average of the four imaging time points over 15 min and the average of both the left and right legs. Data were analyzed by repeated measure analysis of variance (ANOVA). For ANOVA, data were converted to log (base e) scale for the average of max radiance for each treatment group. For wire strength tests and biochemical luciferase assays, data were analyzed by *t*-test, with *P* < 0.05 considered statistically significant.

## Results

### Bioluminescence is detectable in muscles from cardiotoxin-injured *Cox2*^*FLuc/+*^ mice

**Figure 1:**
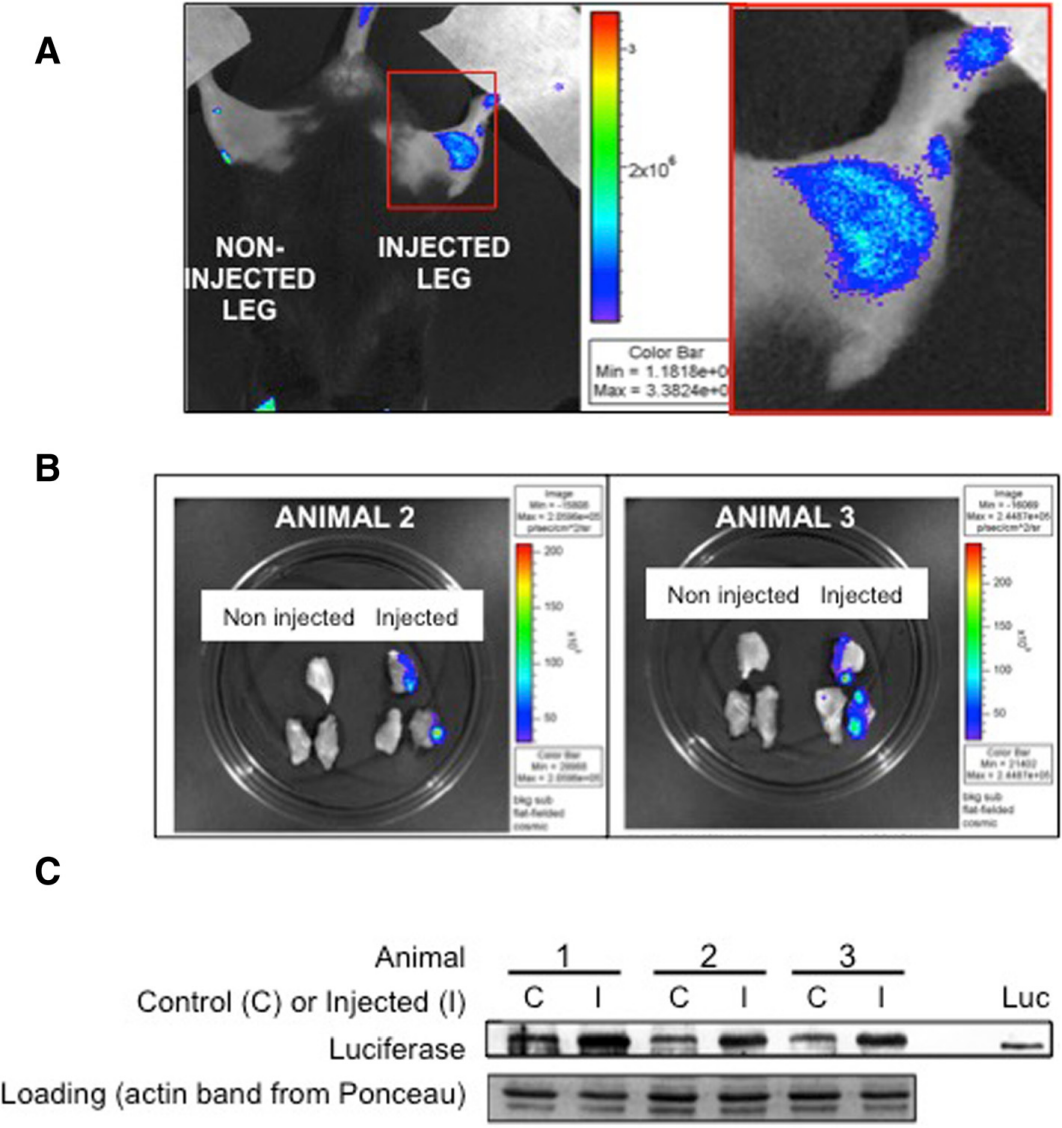
Damaged *Cox2* ^*FLuc/+*^ *DMD* ^*+/+*^ muscle exhibits a bioluminescent signal that is detectable by optical imaging. Optical imaging was carried out on mice injured by cardiotoxin to determine whether the bioluminescent signal induced by muscle damage was detectable. Acute injury was induced by cardiotoxin injection into the lower leg muscles of *Cox2* ^*FLuc/+*^ *DMD* ^*+/+*^ mice while the opposing leg muscles were not injected. Mice were imaged 48 h post cardiotoxin injection. **(A)** *In vivo* bioluminescent signal is shown in pseudo color for one representative mouse. Bioluminescence was observed from the cardiotoxin-injured leg but not from the uninjected leg. Red box in the left panel is shown in higher magnification on the right side of the figure. **(B)** After the first imaging session, the mice were injected again with D-luciferin and the gastrocnemius and hamstring muscles from the left and right legs were dissected, placed in a petri dish, and re-imaged *ex vivo*. Bioluminescence is shown, in pseudo color, for two of the three mice. Only muscle sections from the cardiotoxin-injected legs produced a bioluminescent signal detected by optical imaging. **(C)** Western blot of luciferase detected in whole muscle extracts from uninjected (C) and injected (I) muscles. (Luc) is the luciferase control (recombinant protein) run on the same gel. The actin band from the ponceau red stained nitrocellulose membrane was used as a loading control.

Muscle injury studies were carried out to determine whether the luciferase signal generated by the *Cox2* promoter in *Cox2*^*FLuc/+*^ mice was sufficiently strong to be detectable by optical imaging. To induce injury, cardiotoxin was injected to the area of the leg containing the gastrocnemius and hamstring muscles in three mice. One hind leg of each *Cox2*^*FLuc/+*^ mouse (on a mixed C57B6/B10 wild-type background) was injected with cardiotoxin. The opposing muscle of each mouse was not injected and served as a control. Forty-eight hours after injection the mice were anesthetized, injected with luciferin, and subjected to optical imaging. The injected leg showed a bioluminescent signal, while the uninjected leg did not give a bioluminescent signal. Representative data from one mouse are shown in Figure 1A. Since male mice had a non-specific signal, presumably from the genitalia, we determined that only female mice could be used for these experiments. After imaging was completed, the mice were injected again with D-luciferin. Ten minutes later, the muscles from the left and right hind legs were harvested and imaged *ex vivo* in a petri dish (Figure 1B). Because it was difficult to precisely inject specific muscles with the cardiotoxin, both gastrocnemius and hamstring muscles of the leg were dissected for imaging. For two of the animals, only one of the two pieces of dissected, cardiotoxin-injected hamstring muscle produced a bioluminescent signal, while none of the muscle sections from the uninjected legs, imaged under the same circumstances, showed a signal. It is likely that cardiotoxin did not penetrate all sections of the muscles in the injected leg; the likely explanation for why only two of the three sections showed a signal. Muscles from the three mice were also assessed for luciferase expression by western blotting, confirming the increase in luciferase protein following injury (Figure 1C). Thus, these data suggest that detection of muscle damage *in vivo* by optical imaging is achievable using *Cox2*^*FLuc/+*^ mice.

### Generation of *Cox2*^*FLuc/+*^*DMD*^*−/−*^ mice for optical imaging of dystrophic lesions

The above studies validated that the *Cox2*^*FLuc*^ reporter can generate a detectable optical signal following muscle damage. We next sought to use this reporter to create a dystrophic model to detect muscle inflammation and damage by non-invasive optical imaging. To generate the model, the *Cox2*^*FLuc/+*^ mouse was crossed to the *mdx* (*DMD*^*−/−*^) mouse (hereafter referred to as *Cox2*^*FLuc/+*^*DMD*^*−/−*^ mice) and the *Cox2*^*FLuc*^ transgene was maintained in the heterozygous state. To assess background signal, *Cox2*^*FLuc/+*^ mice on the WT DMD^+/+^ background (*Cox2*^*FLuc/+*^*DMD*^*+/+*^ mice) were imaged in parallel.

### Optical imaging of the *Cox2*^*FLuc*^ reporter detects damage in dystrophic muscle

**Figure 2:**
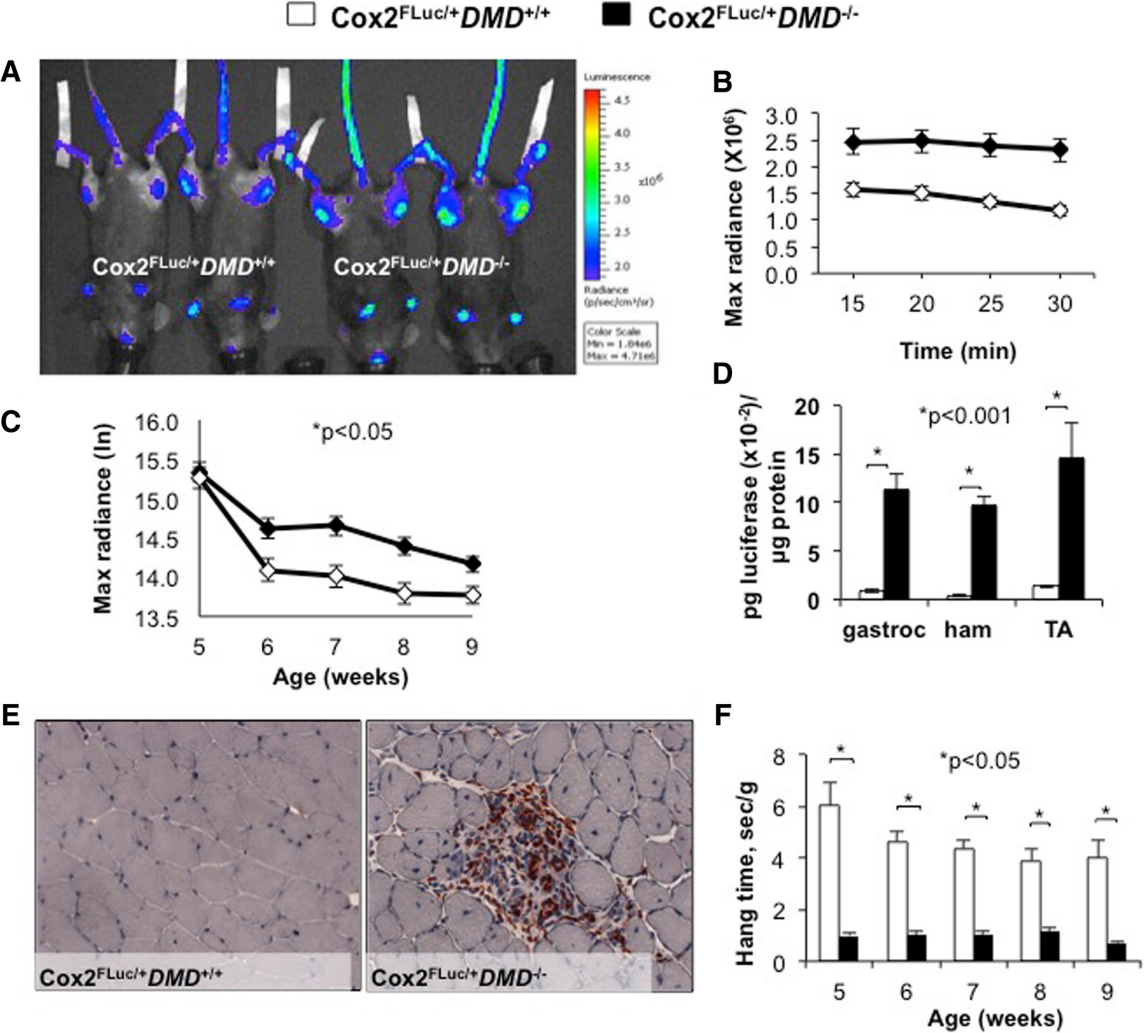
Optical imaging detects muscle damage in exercised *Cox2* ^*FLuc1/+*^ *DMD* ^*−/−*^ mice. **(A)** Representative bioluminescent image taken 25 min after D-luciferin injection into 6-week-old female exercised *Cox2* ^*FLuc/+*^ *DMD* ^*−/−*^ mice and non-exercised *Cox2* ^*FLuc/+*^ *DMD* ^*+/+*^ mice. **(B)** Representative example of data obtained from a group of mice imaged at 6 weeks of age, demonstrating the stability of the bioluminescent signal over the imaging period. (*Cox2* ^*FLuc/+*^ *DMD* ^*+/+*^ *N* = 14, *Cox2* ^*FLuc/+*^ *DMD* ^*−/−*^ *N* = 17) **(C)** The optical imaging data are graphed as log base e (*Cox2* ^*FLuc/+*^ *DMD* ^*+/+*^ *N* = 15, 14, 14, 15, 24, and *Cox2* ^*FLuc/+*^ *DMD* ^*−/−*^ *N* = 18, 17, 19, 20, 35) at 5, 6, 7, 8, and 9 weeks of age. **(D)** *Ex vivo* luciferase enzymatic assays of gastrocnemius, hamstring, and tibialis anterior muscles harvested from *Cox2* ^*FLuc*/+^DMD^−/−^ (*N* = 6) mice and *Cox2* ^*FLuc*/+^DMD^+/+^ (*N* = 3) mice. Mice were 6 to 9 weeks of age. **(E)** Representative cross sections of 8 week old *Cox2* ^*FLuc /+*^ *DMD* ^*+/+*^(left) and *Cox2* ^*FLuc*/+^DMD^−/−^ (right) muscles, stained with a macrophage marker CD68 (red) and counterstained with hematoxylin (blue). **(F)** Muscle strength testing in *Cox2* ^*FLuc /+*^ *DMD* ^*−/−*^ mice (*N* = 9, 15, 11, 11, and 11) and *Cox2* ^*FLuc*/+^DMD^+/+^ mice (*N* = 7, 6, 6, 6, and 5) at 5, 6, 7, 8, and 9 weeks of age.

To evaluate whether the *Cox2-*luciferase signal arising from dystrophic lesions can be detected by optical imaging, we imaged *Cox2*^*FLuc/+*^*DMD*^*−/−*^ mice and age-matched *Cox2*^*FLuc/+*^*DMD*^*+/+*^ mice from ages of 5 to 9 weeks. To exacerbate muscle damage, *Cox2*^*FLuc/+*^*DMD*^*−/−*^ mice were provided running wheels at all times and were also exercised on a treadmill for 20 min, 2 days prior to imaging. *Cox2*^*FLuc/+*^*DMD*^*+/+*^ mice were not exercised, so the background signal emanating from the transgene could be determined. The luciferase signal was best visualized on the quadriceps and hamstring muscles when mice were lying with the ventral surface facing up (Figure 2A). For each mouse, a region of interest on the hindlimb was circled and bioluminescence measured. The signal was stable from 15 to 30 min following luciferin injection (Figure 2B). Mice were imaged once weekly, over a period of 5 weeks (5 to 9 weeks of age) (Figure 2C). A significant difference could be observed between *Cox2*^*FLuc/+*^*DMD*^*−/−*^ (damaged muscle) and *Cox2*^*FLuc/+*^*DMD*^*+/+*^ (healthy muscle) from 6 to 9 weeks of age, by analysis of variance analyses. *Cox2*^*FLuc/+*^*DMD*^*−/−*^ exercised mice showed approximately 40% higher Max Radiance at the time points between 6 and 8 weeks of age, compared to age-matched, non-exercised *Cox2*^*FLuc/+*^*DMD*^*+/+*^ mice (Figure 2C).

Luciferase expression *ex vivo* was assayed in the hamstring, gastrocnemius, and tibialis anterior muscles using a luciferase assay kit. As observed *in vivo*, muscles from *Cox2*^*FLuc/+*^*DMD*^*−/−*^ mice showed significantly higher luciferase expression compared to those from *Cox2*^*FLuc/+*^*DMD*^*+/+*^ mice (Figure 2D). We observed a much larger differential signal between *DMD*^*+/+*^ and *DMD*^*−/−*^ genotypes when luciferase was assayed biochemically compared to assay by optical imaging. The likely reason for this difference relates to the limited resolution of optical imaging of whole muscle. While the resolution of optical imaging is a limitation of this technique, the ability to obtain insight into the status of muscle inflammation in a non-invasive, repetitive, and localized manner counterbalances this limitation to some extent. The ability to assay luciferase at the conclusion of the experiment provides an additional measurement to quantify muscle inflammation and adds to the utility of this mouse model. Comparison of the optical imaging and luciferase data for the last measurement for each mouse provides some degree of comparison for the *in vivo* and *ex vivo* evaluations of *Cox2* promoter-driven gene expression.

Functional and histological studies were also carried out on *Cox2*^*FLuc/+*^*DMD*^*−/−*^ mice to ensure that the mice maintained a dystrophic phenotype. To verify that *Cox2*^*FLuc/+*^*DMD*^*−/−*^ muscles showed damage that is typically seen in *DMD*^*−*^*/*^*−*^ muscles, cross sections of *Cox2*^*FLuc/+*^*DMD*^*−/−*^ muscles were stained for macrophages, and visual inspection confirmed the presence of muscle damage (Figure 2E). To evaluate muscle strength, conventional wire test assays were conducted. *Cox2*^*FLuc/+*^*DMD*^*−/−*^ mice and *Cox2*^*FLuc/+*^*DMD*^*+/+*^ mice were strength-tested once weekly from 5 to 9 weeks of age. Forty-eight hours prior to performing the functional tests, *Cox2*^*FLuc/+*^*DMD*^*−/−*^ mice were exercised on a treadmill for 20 min to exacerbate muscle damage. Similar to *mdx* mice [12], *Cox2*^*FLuc/+*^*DMD*^*−/−*^ mice showed muscle weakness by wire test compared to age-matched *Cox2*^*FLuc/+*^*DMD*^*+/+*^ mice (Figure 2F).

### Identification of cell types expressing the *Cox2*^*FLuc*^ reporter in muscle cross sections

**Figure 3:**
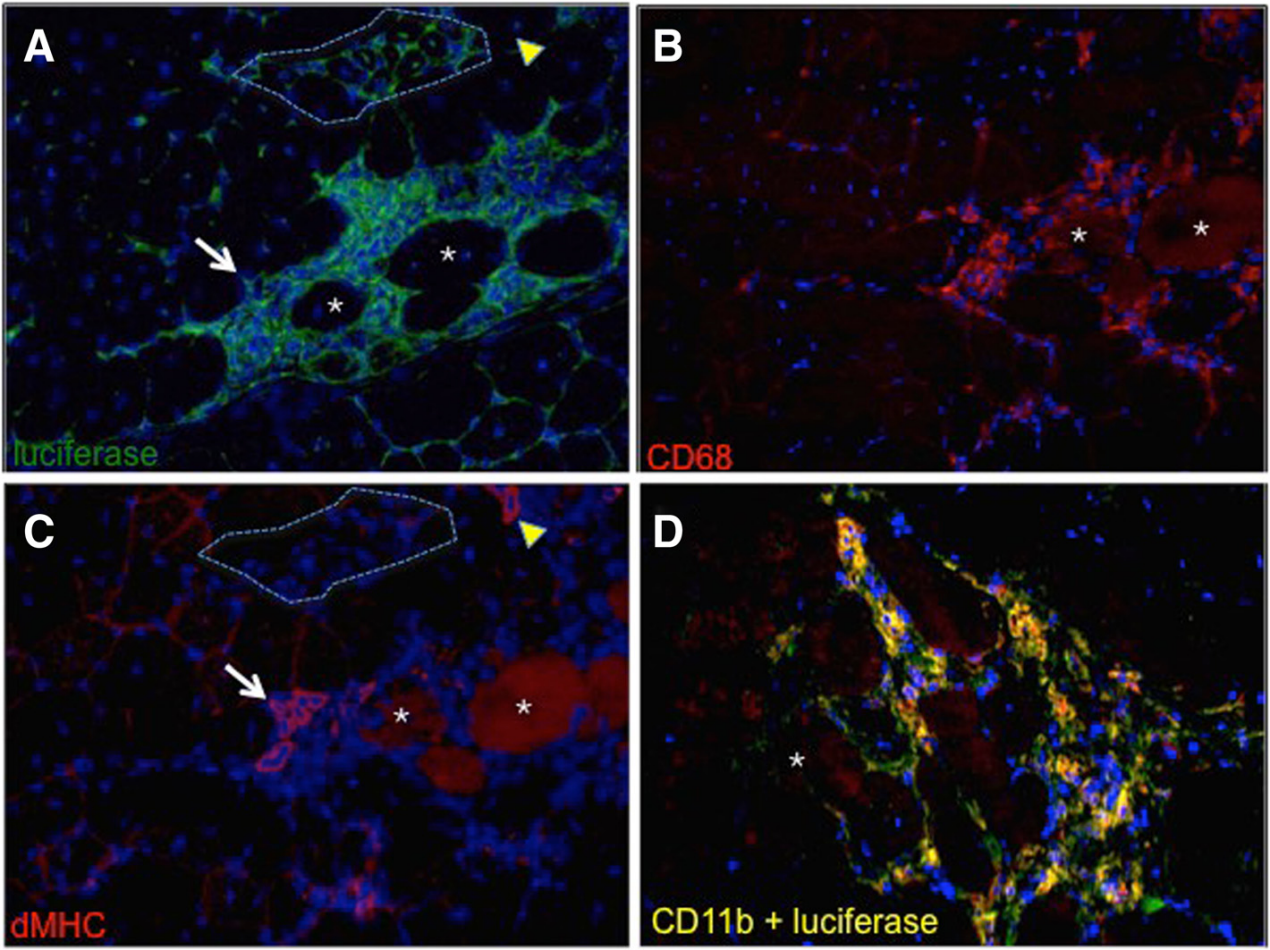
Luciferase signal colocalizes to CD68+ and CD11b + immune cells in *Cox2* ^*FLuc/+*^ *DMD* ^*−/−*^ muscle cross sections. Cross sections of *Cox2* ^*FLuc*/+^DMD^−/−^ mouse quadriceps, fluorescently labeled with antibodies to luciferase (**(A)** and **(D)**, in green), CD68 (red) **(B)**, developmental myosin heavy chain (dMHC) (red) **(C)**, and CD11b (red) **(D)**. **(A)**, **(B)**, and **(C)** are serial sections in which asterisks indicate the same fibers in each of the three sections. The yellow arrowheads in A and C show a regenerating fiber that is dMHC positive but luciferase negative. Similarly, the white arrow points to a patch of regenerating fibers that are luciferase negative. The area outlined by the dotted lines in panels **(A)** and **(C)** shows a cluster of late regenerating fibers that are both dMHC negative and luciferase negative. The micrograph in **(D)** shows a muscle lesion double-labeled with luciferase (green) and CD11b (red, a marker for immune cells). Double stained areas of panel **(D)** appear as yellow. All cross sections were counterstained with DAPI (blue) and were photographed at 20× magnification.

Muscle cross sections from *Cox2*^*FLuc/+*^*DMD*^*−/−*^ mice were stained with a luciferase antibody to validate the specificity of the luciferase signal and to identify the cellular source of luciferase expression. The luciferase antibody signal co-localized with areas that were morphologically distinguishable as dystrophic lesions, corroborating the conclusion that *Cox2* promoter-driven luciferase is a valid indicator of muscle pathology (Figure 3). A robust luciferase signal (shown in green (Figure 3A, D)) co-localized with markers of immune cells; both the M1 macrophage marker CD68 (red color, Figure 3B) or the pan-immune marker CD11b (red color, Figure 3D) co-localized with the luciferase signal (yellow color).

Since small regenerating developmental myosin heavy chain (dMHC+) muscle fibers co-localize to dystrophic lesions, we sought to determine whether these newly developing fibers might also express luciferase. To assess the luciferase signal from regenerating fibers, we compared serial sections stained by either luciferase or dMHC. Comparison of fibers that stained positively for dMHC (Figure 3C) did not reveal a correspondingly strong luciferase signal (Figure 3A) in the same dMHC+ cells (compare red stained structure denoted by yellow arrowhead in Figure 3C to yellow arrowhead in corresponding section of 3A. Similarly, compare structures denoted by white arrows in Figure 3A compared to 3C). Fibers that were regenerating, but no longer expressing dMHC, were clearly negative for luciferase expression (see the area circled by a dotted line in panels A and C of Figure 3). Thus, these studies verify that the signal emanating from muscles of *Cox2*^*FLuc/+*^*DMD*^*−/−*^ mice localizes to dystrophic lesions and derives from immune cells.

### Therapeutic benefit of anti-inflammatory steroids is quantifiable by optical imaging

Treatment with anti-inflammatory steroids is standard of care for patients with DMD, due to the ability of these drugs to extend ambulation in boys with DMD by 3 to 5 years. *Mdx* mice treated with the steroid prednisolone demonstrate initial strength benefits but develop more advanced cardiac fibrosis than non-treated *mdx* mice after 100 days [17]. While steroid treatment of mice does not replicate all the physiological benefits of steroid treatment in humans, the short-term benefit provides a good model to assess the sensitivity of optical imaging to early changes in muscle inflammation.

**Figure 4:**
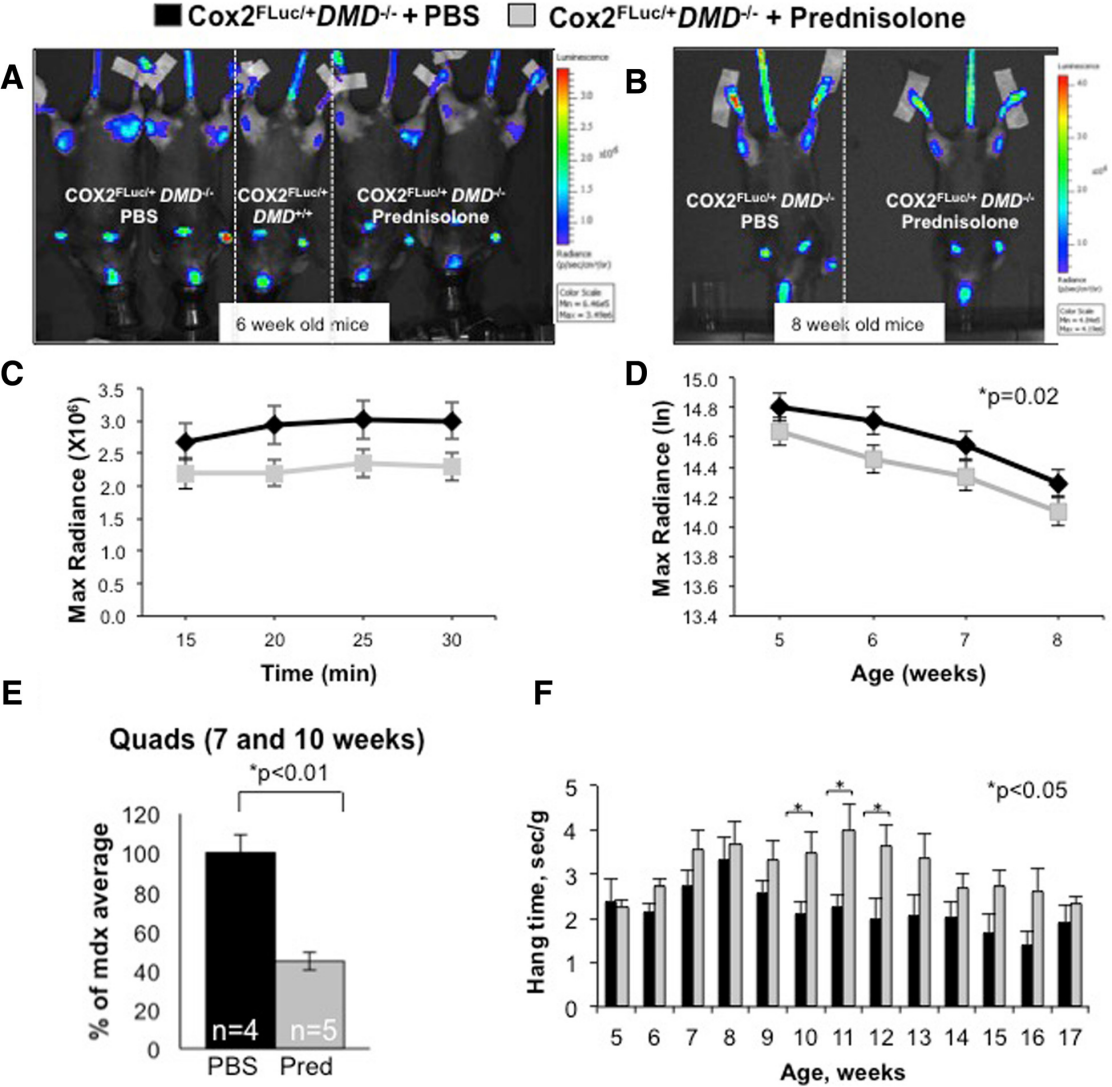
Bioluminescent signal is reduced in prednisolone-treated *Cox2* ^*FLuc/+*^ *DMD* ^*−/−*^ mice. Mice were treated with 0.75 mg/kg of prednisolone or with PBS for 5 days a week starting at 2 weeks of age. Imaging was initiated at 5 weeks of age. **(A)** Representative bioluminescent images taken 25 min after D-luciferin injection into prednisolone-treated *Cox2* ^*FLuc*/+^DMD^−/−^ mice at 6 weeks of age (two mice, far right), PBS-treated *Cox2* ^*FLuc*^ */+DMD−/−* mice (two mice, far left), a control *Cox2* ^*FLuc*/*+*^ *DMD* ^*+/+*^ mouse (one mouse, middle), and **(B)** representative images from mice imaged at 8 weeks of age (PBS treated on left, prednisolone treated on right). All mice were females. **(C)** Representative example of one imaging time point (6 weeks of age) showing the stability of the signal over the 30 min imaging period. (Prednisolone, *N* = 37; PBS *N* = 39). **(D)** Optical imaging data from prednisolone (*N* = 35, 37, 38, 34) and PBS (*N* = 33, 39, 37, 37) treated *Cox2* ^*FLuc*/*+*^ *DMD* ^*−/−*^ mice from 5 to 8 weeks of age. Bars represent standard errors. Data are expressed as max radiance and graphed as log base e, **(E)** luciferase assays of quadriceps muscles harvested from 7 to 10-week-old prednisolone and PBS-treated *Cox2* ^*FLuc*/*+*^ *DMD* ^*−/−*^ mice. **(F)** Wire strength test of prednisolone-treated (*N* = 29, 24, 21, 16, 17, 16, 12 15, 11, 9, 13, 7, and 7) and age-matched control (*N* = 25, 29, 22, 17, 17, 13, 16, 16, 14, 13, 16, 5, and 9) *Cox2* ^*FLuc/+*^ *DMD* ^*−/−*^ mice for ages 5, 6, 7, 8, 9, 10, 11, 12, 13, 14, 15, 16, and 17 weeks. *P < 0.05; PBS, phosphate-buffered saline.

To address the question of whether optical imaging could detect modulation of muscle inflammation after steroid treatment, *Cox2*^*FLuc/+*^*DMD*^*−/−*^ mice were treated with 0.75 mg/kg of prednisolone 5 days a week (starting at 2 weeks of age); control *Cox2*^*FLuc/+*^*DMD*^*−/−*^ mice were injected with an equal volume of PBS. Mice were imaged weekly from 5 to 12 weeks of age. Two days prior to imaging, all mice were exercised for 20 min downhill on a treadmill. Prednisolone significantly reduced the luciferase signal in 5 to 8-week-old mice, when compared to PBS-treated *Cox2*^*FLuc/+*^*DMD*^*−/−*^ mice (Figure 4A, B, C, D). ANOVA revealed a reduction in bioluminescent signal when mice were imaged at 5, 6, 7, and 8 weeks of age following prednisolone treatment. While there was a trend towards a reduced signal in the prednisolone vs PBS-treated muscles at all of the time points between 5 to 8 weeks of age, post hoc analysis revealed statistical significance at the 6-week time point and after all mice from the 5- to 8-week time points were analyzed together. After 8 weeks, differences in signal could not be demonstrated between the treatment groups.

*Ex vivo* luciferase activity was assayed in quadriceps muscles of mice from 7 to 10-week-old treated *Cox2*^*FLuc/+*^*DMD*^*−/−*^ mice, compared to *Cox2*^*FLuc/+*^*DMD*^*−/−*^ mice injected with PBS; prednisolone treatment resulted in a significant reduction in luciferase activity in quadriceps muscles from mice that were 7 to 10 weeks old (Figure 4E) (*P* = 0.002). Similar to the findings presented in Figure 2, biochemical assessment of luciferase by *ex vivo* luciferase enzymatic assay appeared to be a useful indicator of disease activity, even after the difference in treatment groups by luciferase optical imaging was no longer distinguishable.

Muscle strength assays did not detect a difference in strength between treated and untreated *Cox2*^*FLuc/+*^*DMD*^*−/−*^ mice until 10 to 12 weeks of age (Figure 4F). Thus, *Cox2* promoter-driven luciferase optical imaging detected the earlier, anti-inflammatory effects of prednisolone treatment in the muscle prior to the ability to measure functional improvements in muscle strength. These studies demonstrate that the *Cox2*^*FLuc/+*^*DMD*^*−/−*^ mouse is useful for non-invasive detection of early stages of dystrophic disease and for therapeutic modulation of inflammation at the early stages of disease (5 to 8 weeks). The mouse model is also useful for biochemically quantifying *Cox2* promoter-driven luciferase as an evaluation of inflammation in whole muscles in mice 7 to 10 weeks of age Thus, this model provides the opportunity for both non-invasive and biochemical assessment of inflammation in dystrophic muscles. These data validate the *Cox2*^*FLuc/+*^*DMD*^*−/−*^ mouse as a useful model to detect and monitor muscle inflammation, both non-invasively and biochemically.

## Discussion

Translation in muscular dystrophy research has been hindered by a scarcity of reproducible, quantitative outcome measures to assess disease severity in mouse models. While the *mdx* mouse is a valid model of DMD, this mouse must be used in an informed manner to obtain accurate and interpretable data that enables identification of drugs that have the potential to attenuate the clinical disease course in humans [5,8,10]. Despite the utility of the *mdx* model, there is a need for new models that are useful for assessing pharmacological interventions, especially models that allow for data to be collected repeatedly from the same mouse, in longitudinal studies.

In this study we sought to create a mouse model that would allow researchers to carry out non-invasive, repeated, longitudinal assessment of therapeutic interventions and to provide an efficient alternative to conventional analyses of disease progression. When attempting to evaluate improvements in muscle strength following an intervention, dystrophy researchers often use a series of functional assays; these assays, while informative of late aspects of disease progression, are often not useful for assessment of early events in dystrophic disease. For example, while prednisolone treatment of *mdx* mice consistently improves muscular strength, functional benefit cannot be measured until 8 weeks post treatment (Figure 4F). In contrast, non-invasive optical imaging of luciferase expression from the endogenous *Cox2* promoter in *Cox2*^*FLuc/+*^*DMD*^*−/−*^ knock-in mice can detect changes in the inflammatory milieu as early as 3 weeks after the start of prednisolone treatment (Figure 4D). Moreover, additional data on the state of muscle inflammation can also be obtained at sacrifice of the mouse by *ex vivo* monitoring of luciferase activity, increasing the utility of this mouse model.

While currently used methods, such as quantitative histology, may be useful to evaluate drug efficacy, this procedure is labor intensive, subjective, and compromised by sampling bias due to the patchy and asymmetric nature of dystrophic lesions [10]. Optical imaging provides an alternative/supplemental assay to quantitative histology, since multiple muscles are simultaneously sampled and the optical signal is quantified. Moreover, use of tissue sections requires the sacrifice of individual mice at each time point for every analysis, increasing both the uncertainty resulting from inter-animal variability in disease progression as well as differences in pharmacokinetics and pharmacodynamics in individual animals. In contrast, using non-invasive, repeated, longitudinal analysis by bioluminescence imaging in the *Cox2*^*FLuc/+*^*DMD*^*−/−*^ mouse essentially makes each animal its own control. While there is clear and important value in carrying out histological assessments, there is added value in the ability to initially image non-invasively and to then examine the muscle sections in the same animal. Further analysis of luciferase by biochemical assay after the animal is sacrificed provides additional information. Thus, the *Cox2*^*FLuc/+*^*DMD*^*−/−*^ mouse model provides both the ability to non-invasively image aspects of disease progression, followed by traditional histological and luciferase assays.

Two other *mdx* models are available for non-invasive optical imaging. One relies on injection of caged cathepsin compounds into *mdx* mice [18]. This model offers many of the benefits of the model described here. A second model, using the Pax7 promoter to drive luciferase expression, is useful for non-invasively quantifying regeneration [19]. The muscles of this ‘regeneration reporter’ mouse accumulate luciferase signal over the life of the *SJL* mouse (dysferlin-deficient) by perpetually adding nuclei from satellite cells to differentiated muscle. This latter model, when crossed to the *mdx* mouse, would be useful to test potential therapeutic compounds in tandem with the *Cox2*^*FLuc/+*^*DMD*^*−/−*^ mouse to obtain a better evaluation of both early and long-term benefit.

While the *Cox2*^*FLuc/+*^*DMD*^*−/−*^ mouse offers an alternative to other methodologies, there are some limitations to this model. The primary utility of the model is the ability to non-invasively detect inflammation in mice less than 9 weeks of age, when the extent of necrosis is most severe; consequently, its primary value is in assessment of early inflammatory events. Because of the non-invasive aspect of the analysis, multiple time points can be assessed to obtain more extensive data (see Figure 2). If long-term studies are required, luciferase can be quantified biochemically by *ex vivo* enzymatic analysis at the expense of requiring sacrifice of each mouse. We suggest that the *Cox2*^*FLuc/+*^*DMD*^*−/−*^ mouse will be most useful to evaluate therapeutic interventions that impact the immune system. This model will also be valuable to assess interventions that stabilize the muscle membrane, since inflammation is expected to be attenuated following treatments that strengthen the plasma membrane (for example, AAV micro-dystrophin, membrane sealant poloxamer, utrophin upregulation) [20-22]. We suggest that the *Cox2*^*FLuc/+*^*DMD*^*−/−*^ mouse model, which provides both a non-invasive method to assess muscle inflammation in early stages of disease progression and a biochemical alternative to characterize inflammation in later disease stages, will best be used as one of many outcome measures of disease.

## Conclusions

This study describes and validates an inflammation-reporter mouse that is useful for non-invasively and repeatedly assessing therapeutic interventions that reduce muscle inflammation. This model should ultimately be used in conjunction with functional tests, biochemical assessments, and alternative non-invasive imaging models to evaluate the benefit of potential therapeutic interventions.

## Abbreviations

Cox2: cyclooxygenase 2
FLUC: firefly luciferase
PET: positron emission tomography
